# The casein kinase 2 inhibitor, CX-4945, as an anti-cancer drug in treatment of human hematological malignancies

**DOI:** 10.3389/fphar.2015.00070

**Published:** 2015-03-31

**Authors:** Hae J. Chon, Kyoung J. Bae, Yura Lee, Jiyeon Kim

**Affiliations:** Department of Biomedical Laboratory Science, School of Medicine, Eulji University, Daejeon, South Korea

**Keywords:** ALL, AML, CLL, CML, MM, CK2, CX-4945

## Abstract

The casein kinase 2 (CK2) protein kinase is a pro-survival kinase and therapeutic target in treatment of various human cancers. CK2 overexpression has been demonstrated in hematological malignancies, including chronic lymphocytic leukemia, chronic myeloid leukemia, acute lymphoblastic leukemia, acute myeloid leukemia, and multiple myeloma. CX-4945, also known as Silmitasertib, is an orally administered, highly specific, ATP-competitive inhibitor of CK2. CX-4945 induces cytotoxicity and apoptosis and is currently being evaluated in clinical trials for treatment of many cancer types. In the past 2 years, the focus on the therapeutic potential of CX-4945 has shifted from solid tumors to hematological malignancies. CX-4945 exerts anti-proliferative effects in hematological tumors by downregulating CK2 expression and suppressing activation of CK2-mediated PI3K/Akt/mTOR signaling pathways. Furthermore, combination of CX-4945 with other inhibitors yielded synergistic effects in cell death induction. These new findings demonstrate that CK2 overexpression contributes to blood cancer cell survival and resistance to chemotherapy. Combinatorial use of CX-4945 is a promising therapeutic tool for treatment of hematological malignancies.

## Introduction

Among the diverse types of human cancers, hematologic or lymphoid malignancies present major therapeutic challenges due to their low survival rates and poor prognosis. New immuno-chemotherapeutic approaches have improved the survival rates overall; however, many patients with hematologic malignancies, such as chronic lymphocytic leukemia (CLL) and chronic myeloid leukemia (CML), still have poor outcomes due to resistance to chemotherapy, personal therapeutic limits, frequent metastasis, and relapse (Faderl et al., 2009; Gribben and O’Brien, 2011). Thus, there is a need for new and more sophisticated therapeutic strategies for treatment of hematologic malignancies.

The serine/threonine protein kinase, CK2 (casein kinase 2), modulates multiple signaling pathways involved in hematopoietic cell survival and function, and is therefore a promising drug target (Dominguez et al., 2009; Trembley et al., 2009; Piazza et al., 2012). CK2 is constitutively expressed in many cell types. In human cells it typically exists as a tetrameric complex comprising two catalytic alpha subunits (α and α′) and two regulatory (β) subunits (Litchfield, 2003). CK2 plays an important role in the regulation and phosphorylation of a broad range of cellular targets (Pinna, 1990; Litchfield and Lüscher, 1993; Allende and Allende, 1995; Pinna and Meggio, 1997; Guerra and Issinger, 1999; Faust and Montenarh, 2000). In mice, knockout of the CK2α′ subunit induces developmental defects and knockdown of the CK2α and β subunits results in embryonic lethality (Lou et al., 2008; Seldin et al., 2008). CK2 regulates hematopoiesis-associated signaling cascades as well as multiple biochemical processes involving tumor growth, proliferation, and resistance to cytotoxic agents (Piazza et al., 2012). In normal cells, CK2 shows ubiquitous localization throughout the nuclear and cytoplasmic compartments, whereas, in cancer cells, CK2 shows greater abundance in the nuclear compartment (Faust et al., 1999; Laramas et al., 2007). This difference in CK2 distribution may be significant with regard to its biochemical function in cancer. CK2 overexpression has been observed in many hematologic cancers, including CLL (Martins et al., 2010), multiple myeloma (MM; Piazza et al., 2006), T-cell acute lymphocytic leukemia (T-ALL; Silva et al., 2008), and acute myeloid leukemia (AML; Kim et al., 2007; Quotti Tubi et al., 2013). These studies found that CK2α/β mRNA or protein level was increased in cells from several AML patients (approximately 2- to 14-fold more, compared to controls; Quotti Tubi et al., 2013) or from CLL patients (approximately twofold more, compared controls; Martins et al., 2010), as well as plasma cells from MM patients (CK2α, 88% and CK2β, 64% of MM patients analyzed; Manni et al., 2013). Moreover, CK2 upregulation was correlated with poor prognosis (Trembley et al., 2009). These studies identify CK2 as a promising therapeutic target for the development of anti-cancer agents for treatment of many hematological cancers.

## Downregulation of CK2 and Cancer Cell Survival

Overexpression of CK2 has been observed in many cancers, including hematologic cancers such as AML, CLL, T-ALL, and MM (Piazza et al., 2006; Kim et al., 2007; Silva et al., 2008; Martins et al., 2010). Downregulation of CK2, either by transfection of specific siRNA or plasmid-based expression of kinase-inactive CK2, resulted in reduction of cancer cell viability and induction of apoptosis (Faust et al., 2000; Wang et al., 2001; Slaton et al., 2004). Similar to studies addressing non-hematological cancers, RNA interference that targets CK2 was found to induce apoptosis in MM, AML, CLL, and CML (Borgo et al., 2013; Manni et al., 2013; Quotti Tubi et al., 2013; Martins et al., 2014). These reports suggest that downregulation of CK2 by RNA interference or CX-4945 treatment enhances cytotoxicity of hematological cancer cells. Consistent with the overexpression-related observations, these downregulation experiments indicate that CK2 may be a valid druggable anti-cancer target for use in treatment of hematological malignancies, not only human solid tumors (Sarno et al., 2002; Martins et al., 2010; Kim and Kim, 2012).

## CX-4945

CX-4945 (Silmitasertib) is an orally administered, ATP-competitive inhibitor of both CK2α and CK2α′ catalytic subunits that was first developed by Cylene Pharmaceuticals Inc. (Siddiqui-Jain et al., 2010; Pierre et al., 2011b). CX-4945 has been investigated in human cancer studies worldwide and is currently in Phase I and II clinical trials (ClinicalTrials.gov Identifier: NCT02128282). The Phase I trial addresses the safety and tolerability of increasing doses of CX-4945 in combination with gemcitabine plus cisplatin, to determine the maximum tolerated dose (MTD). The subsequent Phase II trial is a randomized study of antitumor activity in cholangiocarcinoma patients, comparing the standard-of-care protocol of gemcitabine plus cisplatin against treatment with CX-4945 in combination with gemcitabine plus cisplatin at the combination MTD determined in the preceding trial.

## Mechanism of CX-4945 Inhibition of CK2

In the molecular model of inhibition, hydrophobic residues in the small and flat ATP binding site of the CK2α subunit can bind ATP or CK2 inhibitors (Sarno et al., 2005). Downregulation of CK2 kinase activity is expected to be due to the ability of inhibitors to establish polar interactions with the active conformation of CK2α. CX-4945 showed a strong interaction with the ATP binding pocket of CK2, with a *K*_i_ = 0.38 [0.02 nM with the recombinant human holoenzyme (ααββ; Ferguson et al., 2011)]. This strong binding interaction between CX-4945 and the ATP binding site of CK2 reduces the enzymatic activity and attenuates the downstream, CK2-regulated PI3K/Akt signaling pathway (Pierre et al., 2011a). The mechanistic relationship between CK2 inhibition by CX-4945, the downstream signaling pathways, and cancer cell survival remains to be fully elucidated.

## The Effect of CX-4945 in Human Lymphocytic/Lymphoblastic Malignancies

The efficacy of CX-4945 has been evaluated with a broad range of human hematologic tumors, including CLL, ALL, AML, and lymphomas (Prins et al., 2013). These studies demonstrated that CX-4945 exerts strong anti-proliferative activity in CLL biopsy samples. As well as decreasing CLL cell viability (IC_50_ < 1 μM) when used alone, CX-4945 exerted synergistic effects in combination with several other inhibitors, including GS-1101, ibrutinib, and fludarabine, which regulate B-cell receptor (BCR)-mediated signaling cascades or downstream mediators. CK2 inhibition downregulates signaling mediators that act downstream of BCR, including PI3K and Akt (Martins et al., 2010, 2011; Ruzzene and Pinna, 2010; Piazza et al., 2012).

In primary CLL cells and in the stable CLL cell line MO1043, CX-4945 treatment led to decreased phosphorylation of Akt and PKC, which are downstream targets of PTEN and PI3K (Martins et al., 2014). Consistent with the *in vitro* effects observed in CLL cells, CX-4945 also showed anti-tumor activity in a mouse xenograft model. CX-4945 treatment caused delayed tumor growth, and treatment with CX-4945 plus fludarabine showed synergistic effects. This pre-clinical evidence suggests that CX-4945 is likely to show therapeutic activity, and that it represents a good candidate for CLL treatment in combination with other anti-tumor agents.

CK2 overexpression is a hallmark of ALL, and two recent studies investigated the relationship between increased CK2 expression and the cytotoxic activity of CX-4945 in T-cell ALL and B-cell ALL (Buontempo et al., 2014; Gomes et al., 2014). CK2 was found to induce phosphorylation of the PTEN tumor suppressor and thereby to activate PI3K/Akt/mTOR, which is a signaling axis that is important for cell survival in ALL (Torres and Pulido, 2001; Vázquez-Franco et al., 2012; Huang et al., 2013; Carnero and Paramio, 2014). CX-4945 treatment resulted in apoptosis of T-cell ALL and B-cell ALL cells (Buontempo et al., 2014; Gomes et al., 2014).

## The Effect of CX-4945 in Human Myeloid Cancers

The therapeutic activity of CX-4945 was also evaluated in CML and AML, respectively. CML is characterized by a translocation known as the “Philadelphia chromosome,” which results in the fusion protein Bcr-Abl, a protein tyrosine kinase that plays a crucial role in cell proliferation and in maintenance of the CML phenotype (Goldman and Melo, 2003). A relationship between Bcr-Abl and CK2 has been previously suggested (Hériché and Chambaz, 1998; Mishra et al., 2003, 2007). Borgo et al. (2013) demonstrated that CX-4945 showed anti-tumor activity in imatinib-resistant CML cells. Downregulation of CK2 by CX-4945 or siRNA contributed to the induction of apoptotic cell death. Furthermore, CK2 inhibition affected the sensitivity of AML cells to chemotherapy. Downregulation of CK2 by CX-4945, K27, or siRNA showed synergistic effects on cytotoxicity and apoptosis in acute, primary blasts as well as in AML cell lines (Quotti Tubi et al., 2013). Moreover, CX-4945 increased the chemotherapeutic activity of daunorubicin in AML.

## Perspective on Combination Therapy with the CK2 Inhibitor, CX-4945, in Hematological Cancers

Inhibition of CK2 expression could also be useful in combination therapies for treatment of MM and mantle cell lymphoma (MCL). A recent report demonstrated CK2 overexpression in MM and MCL cells and that downregulation of CK2 with CK2 inhibitors, such as CX-4945 and K27, induced apoptosis (Manni et al., 2013). Bortezomib, a proteasome inhibitor, exerted anti-tumor activity in MM and MCL cells by stabilization of IκBα in the NF-κB signaling pathway; however, bortezomib alone proved to be insufficient for effective treatment. When used in conjunction with bortezomib, CX-4945 inhibition of CK2 enhanced the cytotoxic activity and mitochondrial-dependent cell death in MM and MCL cells (Manni et al., 2013).

## Conclusion

**FIGURE 1:**
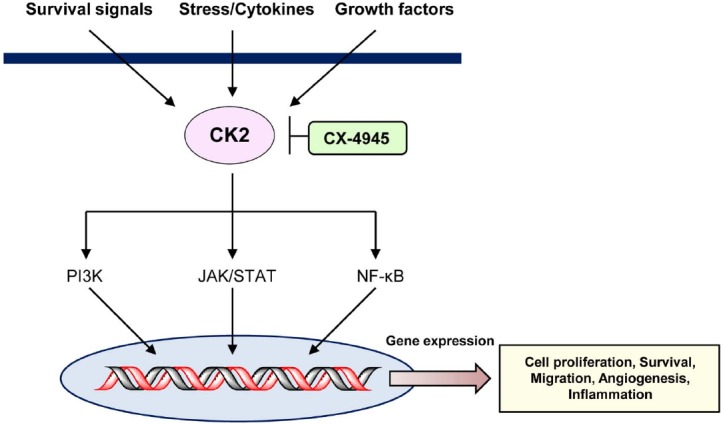
**Schematic of CK2-mediated signaling pathways inhibited by CX-4945**.

Numerous studies have demonstrated the anti-tumor effects of CX-4945 in leukemias or lymphomas, resulting from inhibition of CK2 expression (Figure 1). Based on these results, we propose that CX-4945 has a potential role in novel therapeutic strategies in the future. Additionally, the combination of CX-4945 with various other anti-cancer drugs may be a useful therapeutic strategy for treatment of hematological cancers (Table 1).

**TABLE 1:** **Anti-cancer drugs for potential combination therapy with CX-4945 in treatment of human hematological cancers**.

Disease	Target CK2 subunits	Combined inhibitors	Target	IC_50_ or *K*_i_	Reference
CLL	α, α′	Ibrutinib	BTK (Bruton’s tyrosine kinase)	0.5 nM (*K*_i_)	Honigberg et al. (2010)
ALL	α, α′	Temsirolimus	mTOR	1.76 μM	Shor et al. (2008)
CML	α, β	Imatinib	Bcr-Abl	0.6 μM	Buchdunger et al. (1995)
AML	α, β	Daunorubicin	DNA or RNA synthesis	0.02 μM	Gewirtz (1999)
MM	α, β	Bortezomib	20S proteasome	0.6 nM (*K*_i_)	Adams et al. (1999)

## Author Contributions

HC, KB, and YL collected and analyzed the background research and created the figure and the table. JK wrote the manuscript.

### Conflict of Interest Statement

The authors declare that the research was conducted in the absence of any commercial or financial relationships that could be construed as a potential conflict of interest.
